# Successful closure of a tracheocutaneous fistula after tracheostomy using two skin flaps: a case report

**DOI:** 10.1186/s40792-015-0045-1

**Published:** 2015-05-27

**Authors:** Yui Watanabe, Tadashi Umehara, Aya Harada, Masaya Aoki, Takuya Tokunaga, Soichi Suzuki, Go Kamimura, Kazuhiro Wakida, Toshiyuki Nagata, Tsunayuki Otsuka, Naoya Yokomakura, Kota Kariatsumari, Yoshihiro Nakamura, Yuko Watanabe, Masami Sato

**Affiliations:** Department of General Thoracic Surgery, Graduate School of Medical and Dental Sciences, Kagoshima University, 8-35-1 Sakuragaoka, Kagoshima, 890-8570 Japan; Department of Anesthesiology and Critical Care Medicine, Graduate School of Medical and Dental Sciences, Kagoshima University, Kagoshima, Japan

**Keywords:** Tracheocutaneous fistula, Tracheostomy, Skin flap

## Abstract

A tracheocutaneous fistula may develop when a tracheostomy orifice epithelializes during a prolonged course of healing or undernutrition. Various techniques for closing such fistulae have been reported. However, a standard procedure has not yet been established. We, herein, present a case involving a 35-year-old woman who developed a tracheocutaneous fistula after tracheostomy. We closed the fistula using two skin flaps to cover the tracheal lumen and skin defect, respectively. The advantage of this technique is that it allows the tracheal lumen to be covered by inversed skin epithelium and ensures that the suture line of the skin does not match up with that of the subcutaneous tissue.

## Background

Tracheostomy is a general surgical procedure performed by many thoracic surgeons on a routine basis. A tracheostomy orifice closes by second intention in many routine cases. However, the orifice sometimes epithelializes and develops a tracheocutaneous fistula, especially if the healing course is prolonged or the patient has a poor nutritional status. In such cases, surgery is required to close the fistula. During surgery, it is important to definitively cover both the tracheal and skin defects. Additionally, attention should be paid to avoid promoting postoperative tracheal stenosis secondary to granulation.

## Case presentation

**Fig. 1:**
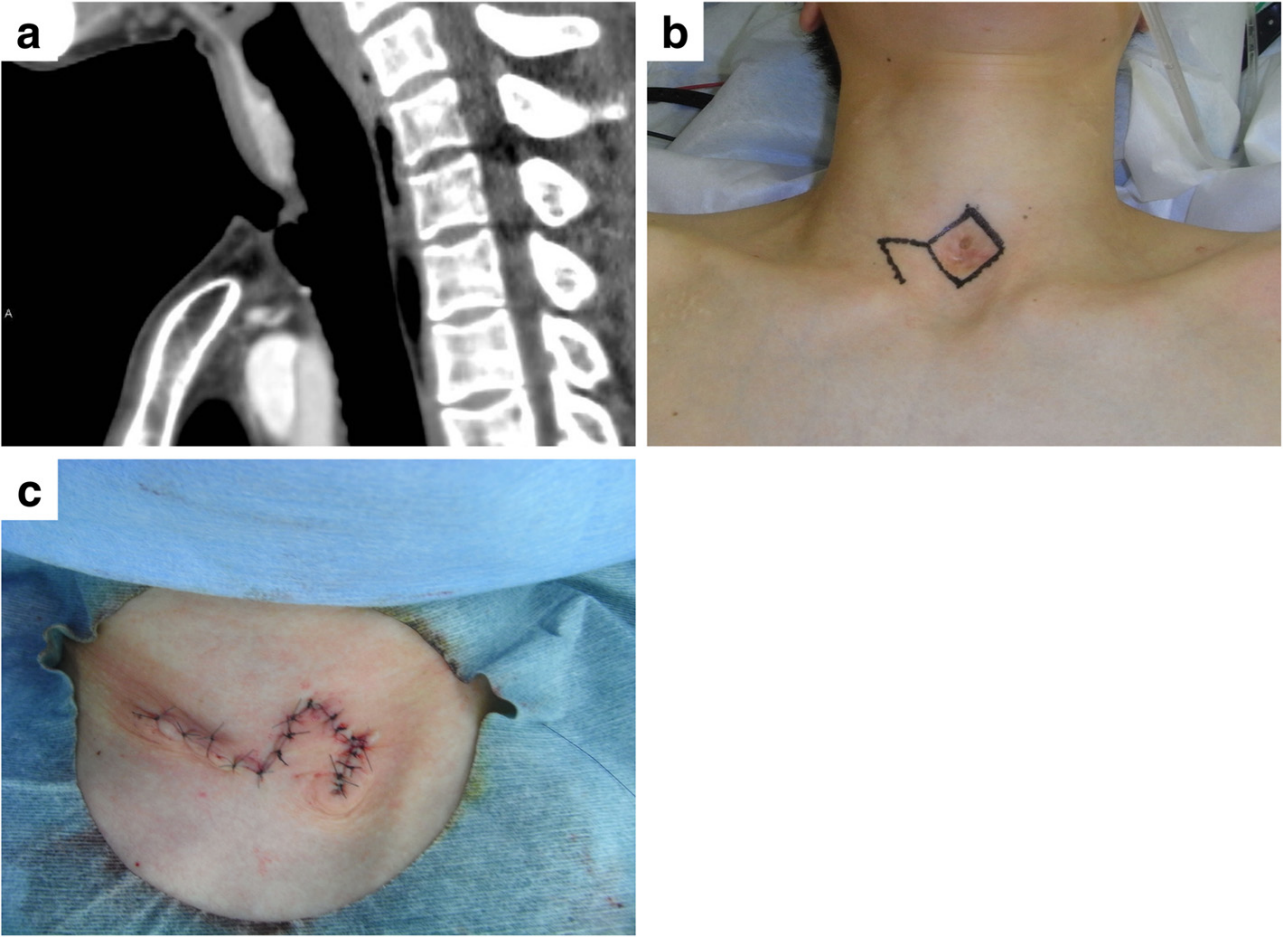
Preoperative and operative findings. **a** Cervical sagittal computed tomography showed a tracheocutaneous fistula. **b** The skin incision was designed as a box around the fistula and two sequential straight lines. **c** A single flap was elevated and rotated to cover the skin defect, and the skin was sutured layer by layer

A 35-year-old woman was scheduled to undergo surgery to close her tracheocutaneous fistula. The patient had previously undergone tracheostomy with an inverted U-shaped tracheal incision and mechanical ventilation for central neurogenic respiratory failure due to autoimmune limbic encephalitis when she was 30 years old. Although she recovered from her previous condition after 11 months of tracheal cannulation, she subsequently developed a tracheocutaneous fistula due to the prolonged course of healing and poor nutritional status. Laboratory analysis revealed that her white blood cell count was 2900/μL, serum total protein level was 5.3 g/dL, and serum albumin level was 3.0 g/dL. Neck sagittal computed tomography showed a tracheocutaneous fistula (Fig. 1a). Although repair of such fistulae is possible under local anesthesia, her surgery was performed under general anesthesia because the encephalitis had caused residual restlessness.

**Fig. 2:**
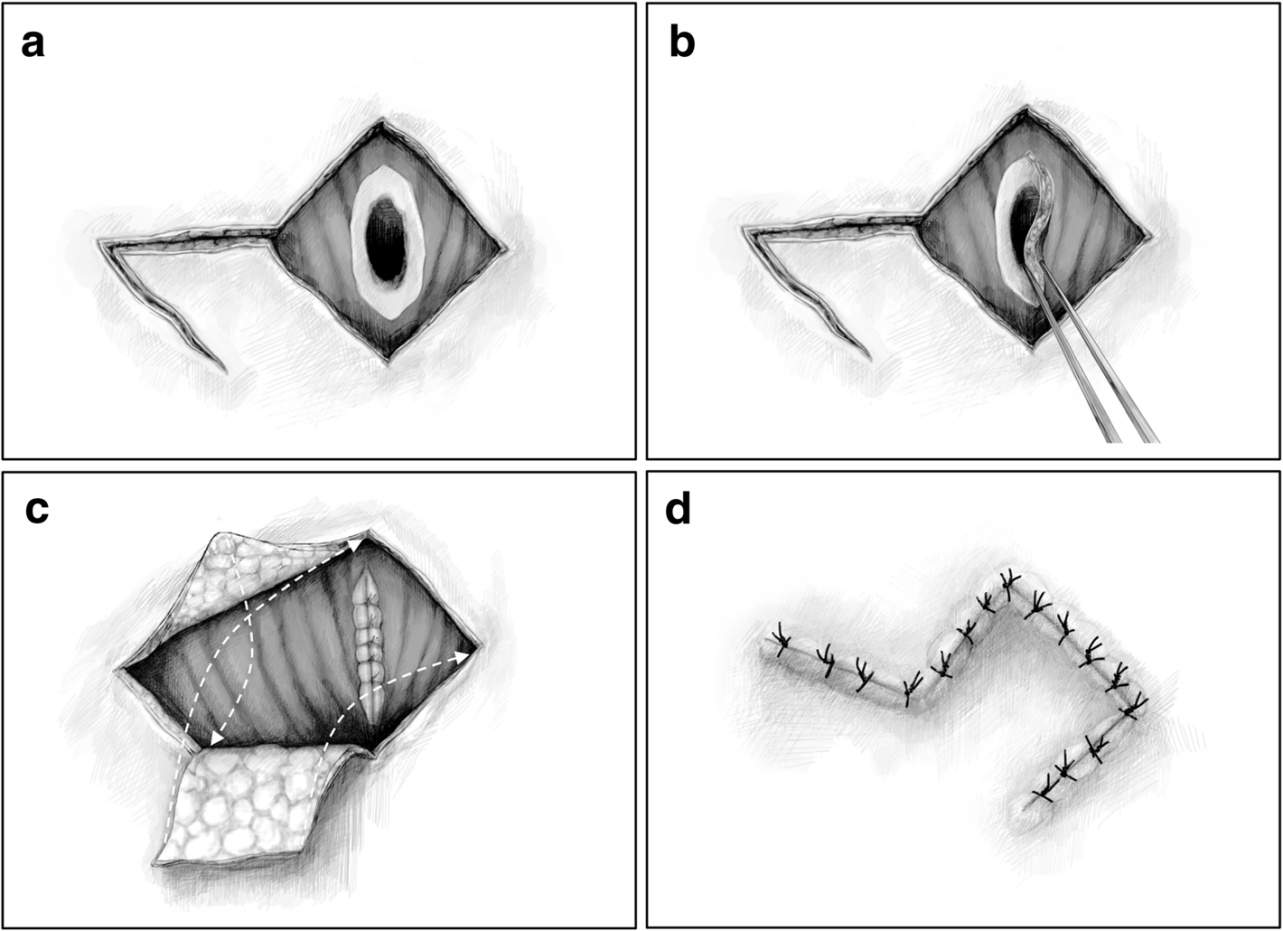
Operative schemes. **a** Bilateral hinge flaps were created to close the fistula. The flaps were taken from the left and right sides of the tracheal defect, and the superfluous skin of each hinge flap was trimmed. **b** The hinge flaps were inversed to cover the tracheal lumen with skin epithelium and sutured tightly with the platysma muscle. **c** A single flap to cover the skin defect was elevated from the lateral aspect of the right hinge flaps. The *arrows* indicate how the skin flaps were positioned. **d** The single flap was elevated and rotated to cover the skin defect, and the skin was sutured layer by layer

The skin incision was designed as a box around the fistula with two sequential straight incisions emanating from one corner (Fig. 1b). The length of each skin incision was 25 mm. Bilateral hinge flaps to close the fistula were created from the left and right sides of the tracheal defect, and superfluous skin from each hinge flap was trimmed (Fig. 2a). A single flap covering the skin defect was elevated from the lateral aspect of the right hinge flap to join with the corresponding sides of the defect (Fig. 2b). The hinge flaps were inversed to cover the tracheal lumen with skin epithelium and sutured tightly with the platysma muscle using absorbent strings (Fig. 2b). The elevated and rotated single flap covered the skin defect, and nonabsorbent suture was used to close the skin in a layer-by-layer fashion to avoid creating dead space (Figs. 1c and 2c). The operative time was 58 min, and a drainage tube was not placed. Her postoperative course was uneventful, and she had no recurrence of the tracheocutaneous fistula for 18 months.

Several procedures to close tracheocutaneous fistulae have been previously reported [1–7]. However, no standard, easily performed procedure has been established worldwide. One advantage of the procedure described herein is that postoperative tracheal stenosis secondary to granulation is not likely to occur because the tracheal lumen is covered by inversed skin epithelium. The bilateral hinge flaps closing the tracheal defect can be lifted from not only the left and right sides but also from the upper and lower sides depending on the form of the defect. Moreover, the superfluous skin of each hinge flap can be trimmed to create appropriately sized flaps. Another advantage is that recurrence of the tracheocutaneous fistula after wound dehiscence is not likely to occur because the suture line of the skin cannot overlap with that of the subcutaneous tissue. Most patients can undergo this procedure with local anesthesia. This technique for closing tracheocutaneous fistulae after tracheostomy using two skin flaps is thus considered to be beneficial.

## Conclusions

We have presented a case of successful closure of a tracheocutaneous fistula after tracheostomy using two skin flaps. This technique has two advantages: (1) it allows the tracheal lumen to be covered by inversed epithelium and (2) it ensures that the suture line of the skin does not match up with that of the subcutaneous tissue.

## Consent

Written informed consent was obtained from the patient for publication of this case report and any accompanying images. A copy of the written consent is available for review by the Editor-in-Chief of this journal.

## Abbreviation

CT: computed tomography
